# Change in Color and Volatile Composition of Skim Milk Processed with Pulsed Electric Field and Microfiltration Treatments or Heat Pasteurization ^†^

**DOI:** 10.3390/foods3020250

**Published:** 2014-04-23

**Authors:** Anupam Chugh, Dipendra Khanal, Markus Walkling-Ribeiro, Milena Corredig, Lisa Duizer, Mansel W. Griffiths

**Affiliations:** 1 Department of Food Science, University of Guelph, Guelph, ON N1G 2W1, Canada; E-Mails: achugh@uoguelph.ca (A.C.); dkhanal@uoguelph.ca (D.K.); mcorredi@uoguelph.ca (M.C.); lduizer@uoguelph.ca (L.D.); mgriffit@uoguelph.ca (M.W.G.); 2 Department of Food Science, Cornell University, Ithaca, NY 14853, USA

**Keywords:** pulsed electric field (PEF), microfiltration (MF), thermal pasteurization, skim milk, volatile compounds, color degradation, non-thermal processing, hurdle technology

## Abstract

Non-thermal processing methods, such as pulsed electric field (PEF) and tangential-flow microfiltration (TFMF), are emerging processing technologies that can minimize the deleterious effects of high temperature short time (HTST) pasteurization on quality attributes of skim milk. The present study investigates the impact of PEF and TFMF, alone or in combination, on color and volatile compounds in skim milk. PEF was applied at 28 or 40 kV/cm for 1122 to 2805 µs, while microfiltration (MF) was conducted using membranes with three pore sizes (lab-scale 0.65 and 1.2 µm TFMF, and pilot-scale 1.4 µm MF). HTST control treatments were applied at 75 or 95 °C for 20 and 45 s, respectively. Noticeable color changes were observed with the 0.65 µm TFMF treatment. No significant color changes were observed in PEF-treated, 1.2 µm TFMF-treated, HTST-treated, and 1.4 µm MF-treated skim milk (*p* ≥ 0.05) but the total color difference indicated better color retention with non-thermal preservation. The latter did not affect raw skim milk volatiles significantly after single or combined processing (*p* ≥ 0.05), but HTST caused considerable changes in their composition, including ketones, free fatty acids, hydrocarbons, and sulfur compounds (*p* < 0.05). The findings indicate that for the particular thermal and non-thermal treatments selected for this study, better retention of skim milk color and flavor components were obtained for the non-thermal treatments.

## 1. Introduction

Milk is a widely consumed beverage due to its nutritional importance, a pleasant aroma and mouth-feel, and a slightly sweet taste. Color and flavor are important sensorial attributes of milk that are influenced by several factors, such as milk composition, cow’s feed and metabolism, environmental factors and processing conditions [1,2]. Few studies are available on the impact of conventional thermal treatments and emerging processing technologies like pulsed electric field (PEF), high hydrostatic pressure (HHP) and ultra high pressure (UHP) on volatile compounds in milk or milk based beverages [3,4,5,6]. Pulsed electric field (PEF) and microfiltration (MF) are emerging innovative technologies that could meet the increasing consumer demand for “fresh-like” minimally processed foods. The mode of action that underlies PEF is based on short electric pulses of high voltage applied to a product that is placed between a pair of electrodes, thereby, bringing about electroporation of the bacterial cell wall and its subsequent breakdown [7]. In addition to the type of microorganism and its innate resistance [8], the efficacy of PEF for food preservation depends on processing factors such as electric field strength, number of pulses applied and the treatment time [9], as well as product parameters, including electrical conductivity, viscosity, and pH [10].

MF is a membrane-driven separation process typically employing membranes with a pore size of 1.2 to 1.4 µm that allows removal of bacteria in vegetative and spore forms from milk [11,12]. Commercially available thermal processing methods promise a high degree of microbial safety but can adversely affect other food properties such as color and volatile compounds. Several studies have demonstrated a comparable impact of PEF and MF processes on microbial inactivation in foods [13,14,15,16,17]. Milk has its natural color due to the reflectance of light by dispersed milk fat globules, proteins, and natural milk pigments like riboflavin and carotenoids [18,19]. However, milk color is altered at high processing temperatures as a result of Maillard browning or a temporary increase in lightness due to denaturation of soluble whey proteins [20,21]. The changes in milk compounds that are responsible for change in milk color may, at the same time, affect the perception of flavor in milk due to intensification of some volatile components. It is generally accepted that prolonged or severe heat treatments cause the degradation of the volatile profile of milk, with the increase in concentration of volatile compounds being positively correlated to intensity of the heating [22,23]. The development of such volatile compounds may alter consumer acceptance as suggested by Gandy and others [24]. In contrast, non-thermal processing may have a minimal effect on the concentration of volatiles in foods due to shorter processing times at temperatures below those used for pasteurization. Zhang *et al.* [5] observed increased levels of aldehydes and ketones in pasteurized milk as compared to raw and PEF processed milk. PEF-treated and HHP-treated orange juice-milk beverage showed a reduced loss of volatiles as compared to thermally processed beverage [3]. From a quality and safety perspective, it may be advantageous to use hurdle technology rather than using a single technology to achieve sufficient product safety and maximized quality while maintaining minimal processing. Microfiltration is one of the non-thermal technologies that have been commercially allowed for extending shelf life of milk, although its use as a stand-alone treatment is restricted due to regulatory requirements [25]. Based on microbiological data, previous studies have indicated that the safety of skim milk processed with a combination of PEF and MF is comparable to that achieved by HTST pasteurization [13,17], potentially representing a non-thermal alternative to the well-established heat pasteurization. However, the effect of this novel process on color and volatile compounds has not been determined. Thus, the objective of the present study was to study the effect of different PEF, MF, and hurdle (PEF/MF) processing conditions on the color and flavor profiles of skim milk. In addition, the retention of color and flavor following treatments with these non-thermal technologies was compared to that obtained by heat pasteurization.

## 2. Experimental Materials and Methodology

### 2.1. Supply, Preparation, and Storage Conditions of Skim Milk

Raw milk obtained from a local dairy processing plant was separated at the Canadian Research Institute for Food Safety at the Department of Food Science, University of Guelph, using a cream separator (STsM-100-18, Motor Sich JSC, Zaporozhye, Ukraine). Skim milk was selected for processing in this study due to its lower fat content, allowing efficient microfiltration without membrane blockage, and because it is common research model for whole milk and has grown in consumer popularity.

### 2.2. Pulsed Electric Field (PEF) Treatment of Skim Milk

Raw skim milk processing was carried out in a PEF treatment chamber designed at the University of Guelph using an exponential decay pulse generator (PPS 30, University of Waterloo, Waterloo, ON, Canada) to form monopolar pulses with an average width of 1.5 µs. PEF parameters were measured and monitored as described previously [17]. The single PEF chamber used consists of two co-axial stainless steel electrodes encased in an insulating plastic casing. Liquid passes between electrodes through a gap of 0.21 cm. The chamber has a hold up volume of 44 cm^3^ of which 25 cm^3^ is the volume between the electrodes where milk is treated by means of electric pulses. An external jacket with a volume of 790 cm^3^ surrounds the outer electrode for additional temperature control, allowing the PEF chamber to be heated or cooled during operation. Skim milk was pumped through Masterflex silicone tubing (size L/S 16) using a peristaltic pump (Masterflex pump drive 7524-40 and pump head 77201-60, Cole Parmer Instrument Co., Vernon Hills, IL, USA). The product was pumped at a flow rate of 20 to 35 mL/min and the temperature at the entry and exit of the PEF chamber was recorded using T-type thermocouples (TMTSS-040G-6, Omega, Stamford, CT, USA) connected to a wireless temperature data logger (OM-SQ2020-2F8, Omega, Stamford, CT, USA). After treatment the outgoing product was cooled by flow through tubing submerged in a cooling water bath (NESLAB RTE 7, Thermo Fisher Scientific Inc., Newlington, NH, USA), set at 2.5 °C. For hurdle treatment, involving subsequent microfiltration, samples were kept in ice in a cold room at 4 °C before further processing to maintain the cold chain and thus, avoid growth of mesophilic microorganisms and potential quality degradation in line with common practice used in the dairy industry. Experiments were conducted at three PEF intensities that reflect different processing demands for skim milk with regard to food safety and energy consumption, which can vary considerably, based on production facilities and locations, and have not been investigated for comparable effects on milk quality parameters, such as color and volatile composition, based on these treatment intensities. The skim milk was processed using electric field strength and treatment time combinations of 28 kV/cm and 2805 µs for low-intensity PEF (PEF-L), 40 kV/cm and 1122 µs for moderate-intensity PEF (PEF-M), and 40 kV/cm and 1571 µs for high-intensity PEF (PEF-H). These processing conditions corresponded to energy densities of 83, 157 and 198 kJ/L, respectively. A pulse frequency of 25 Hz was applied for PEF-L, whereas 17.5 Hz were used for PEF-M and PEF-H treatments. Milk temperature at the inlet of the PEF chamber was 17 °C on average and depending on the treatment intensity applied maximum temperatures of 37 (PEF-L), 56 (PEF-M), and 65 (PEF-H) °C were obtained at the outlet. Following PEF and the cooling step the product temperature was 10 °C and samples were collected at this temperature.

### 2.3. Microfiltration (MF) of Skim Milk

Bearing in mind that smaller sized membrane pores allow for enhanced microbial reduction in skim milk, the latter was microfiltered at different pore size diameters to compare their effect on the product quality. Moreover, lab and pilot scale systems featuring different membrane materials and designs were used for MF to determine whether or not the processing scale and/or membrane materials and/or membrane designs affect color and volatile compound composition in skim milk. Tangential-flow microfiltration (TFMF) was performed using a laboratory scale Supor tangential-flow filtration system featuring either 0.65 µm (medium screen, 1.4 cm thick) or 1.2 µm (suspended screen, 1.8 cm thick) pore-sized polyethersulfone membranes (Pall Corporation, Port Washington, NY, USA). For laboratory-scale TFMF, a flow rate of 300 mL/min was generated using a Masterflex EW 77200-60 pump (Cole Parmer Instrument Co., Vernon Hills, IL, USA) and the skim milk was delivered by silicone tubing (Masterflex 96400-25, Cole Parmer Instrument Co., Vernon Hills, IL, USA) through either of the TFMF membrane cassettes, with trans-membrane pressures ranging from 0.3 to 0.6 kPa. The permeate flow rate was between 7 and 13 mL/min for milk passing through the TFMF 0.65 µm membrane (MF-0.65), whereas it was between 37 and 53 mL/min for the TFMF 1.2 µm (MF-1.2) membrane. In addition, skim milk processing was also carried out on a pilot scale 1.4 μm pore size, ceramic membrane, cross-flow microfiltration (CFMF) unit (MFS-1, Tetra Pak Filtration Systems, Aarhus, Denmark). The CFMF unit was used at a permeate flow rate (100 L/h) ten times that of retentate (10 L/h), using filter module Type-SCT Membralox (Tetra Pak Filtration Systems). The trans-membrane pressure of the ceramic membrane module was 106 kPa. All microfiltration treatments were carried out at a constant temperature of 35 °C after pre-heating skim milk in a water bath (Isotemp 210, Thermo Fisher Scientific Inc., Newlington, NH, USA) set to this temperature and equipped with a thermometer. In agreement with membrane and equipment manufacturer specifications milk volumes were selected for TFMF and CFMF processing that allowed proper milk circulation through the membranes and process stability before sampling. To avoid membrane clogging over time, laboratory scale membranes were cleaned by recirculating 0.5 N sodium hydroxide followed by 0.1 N nitric acid and 2% bleach at 50 °C, and a final rinse with water immediately after each run. Following the same approach but using different cleaning agents the pilot plant CFMF ceramic membrane was cleaned by recirculation of commercially available mixed acid detergent descaler (Diversey Divos2 (VM13), Diversey, Oakville, ON, Canada) and caustic detergent (Diversey Liquid Bril Tak (VC85)) followed by a final rinse with water.

### 2.4. PEF/MF Processing of Skim Milk

For hurdle processing, milk was processed using PEF as described above and stored at 4 °C prior to processing with MF. All three PEF treatments: PEF-L, PEF-M and PEF-H were used for hurdle processing followed by MF-0.65 µm and MF-1.2 µm membranes (PEF/MF). However, due to the difficulty in achieving the high sample volumes required for pilot scale microfiltration (MF-1.4) using the laboratory scale PEF unit, only skim milk processed using the PEF-M treatment was coupled with the MF-1.4 µm treatment.

### 2.5. High-Temperature Short-Time Pasteurization of Skim Milk

High-temperature short-time (HTST) processing of raw skim milk was carried out with a pilot scale, dual stage heat exchanger unit (UHT/HTST Lab-25 EDH, Microthermics Inc., Raleigh, NC, USA) at the Guelph Food Technology Centre (GFTC), Guelph, ON, Canada. Skim milk was kept at 4 °C before processing in the tubular heat exchanger. Milk was processed at two different temperatures of 75 (HTST-75) and 95 °C (HTST-95) for respective holding times of 20 and 45 s, corresponding to flow rates of 0.9 and 0.4 L/min, respectively. The higher intensity HTST pasteurization (95 °C, 45 s) for milk takes into consideration an additional safety margin and extended shelf life in part applied by the industry, but at the same time increased thermal load also renders changes in color and volatile compounds more likely. The heat exchanger pre-heated milk to 55 °C prior to reaching the respective temperatures and cooled the product to 10 °C after the treatments and prior to sampling.

### 2.6. Color Analysis

Color attributes were measured in Hunter Lab color space using a CM 3500-d spectrophotometer (Konica Minolta Sensing Inc., Mahwah, NJ, USA) equipped with SpectraMagic NX CM-S 100 software (Konica Minolta Sensing Inc.). Reflectance measurements were collected over a wavelength range from 400 to 700 nm. The color values were expressed in Hunter Lab color space as lightness (*L*), redness (*a*), and yellowness (*b*). In order to compare the total color difference (Δ*E*), between the color properties of untreated samples to those of values obtained after subjecting milk to different treatments, the following equation [26,27] was utilized:

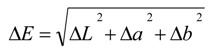
(1)


where, Δ*L* = *L*_standard_ − *L*_sample_, Δ*a* = *a*_standard_ − *a*_sample_, Δ*b* = *b*_standard_ − *b*_sample_.

In addition, the whiteness was calculated for the skim milk samples by converting Hunter Lab to CIE 1931 XYZ color space values using following formulas:

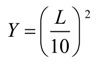
(2)

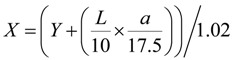
(3)

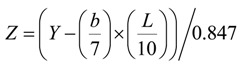
(4)


and then using American Society for Testing and Materials (ASTM) E313 standard practice for calculating Whiteness index (WI):

WI = (3.388 × Z) - (3 × Y)
(5)

For each treatment, samples were collected in triplicate. Color measurements were done in duplicate for each sample.

### 2.7. Analysis of Volatile Compounds

Headspace volatiles in skim milk were analyzed using SIFT-MS (Selected In Flow Tube-Mass Spectrometry), which is a direct mass spectrometer that applies chemical ionization for the analysis of volatile components, yielding product ions that are analyzed with SIFT-MS technology (Voice200^®^, Syft Technologies Ltd., Christchurch, New Zealand). The latter identifies and quantifies volatile compounds with a built-in scientific library, that is experimentally determined with product ions and reaction rate coefficients, in combination with the flow tube geometry, the ionic reaction time, the measured flow rates and the pressure applied during sample analysis and requires no chemical standards for calibration. Skim milk samples (35 mL) were placed in 300 mL glass jars with lids closed and then warmed to room temperature before analysis. An empty jar was used as a blank. Twenty-one flavor compounds associated with milk were quantified using selected ion mode with 100 ms time limit, count limit of 10,000, 30 s background scan time and a product scan time of 60 s. Helium was applied as the carrier gas (250 kPa) and the scan was performed following charge transfer from three positively-charged reagent ions (H_3_O^+^, NO^+^, and O_2_^+^) to the analyte in the flow tube. The temperature of the inlet arm extension was 120 °C and flow tube pressure was 113.6 ± 1.7 mTorr.

Samples were collected in triplicate for each treatment with a duplicate analysis for each sample.

### 2.8. Statistical Data Analysis

Color and flavor data were statistically analyzed with Sigmaplot version 11.0 (Systat Software Inc., London, UK). One-way analysis of variance (ANOVA) was carried out for three different batches of skim milk (*n* = 3), analyzing all treatments at a confidence interval of 95%. When statistical differences between treatment means were observed, the Holm-Sidak method for multiple pairwise comparisons was used. For adequate reproducibility each treatment was repeated three times and analysis was performed in duplicate.

## 3. Results

### 3.1. Color Attributes of Non-Thermally and Thermally Treated Skim Milk

No significant difference in color was found in untreated skim milk and when processed using PEF-L, PEF-M, PEF-H, MF-1.2 and MF-1.4 and different combinations of PEF with MF treatments. The values for the color attributes *L*, *a*, and *b* following various treatments are provided in Table 1.

**Table 1:** Comparison of Hunter color space attributes *L* (lightness), *a* (redness) and *b* (yellowness) measured in raw skim milk and after processing with high-temperature short-time pasteurization at 75 (HTST-75) and 95 (HTST-95) °C, pulsed electric field at low (PEF-L), moderate (PEF-M) and high (PEF-H) intensities, 0.65 and 1.2 μm pore size tangential flow microfiltration (0.65 TFMF and 1.2 TFMF, respectively), 1.4 μm-pore size cross-flow microfiltration (1.4 CFMF), and different pulsed electric field-based combinations with the latter three membrane filtration treatments.

Treatments	*L*	*a*	*b*	Δ*E*
Raw Skim Milk	64.50 (±0.87) ^a^	−3.92 (±0.08) ^a^	−0.15 (±0.15) ^a^	-
HTST-75	65.00 (±0.61) ^a^	−4.01 (±0.10) ^a^	−0.08 (±0.02) ^a^	0.51
HTST-95	65.21 (±0.10) ^a^	−4.03 (±0.01) ^a^	0.19 (±0.04) ^a^	0.79
0.65 TFMF	53.90 (±1.25) ^b^	−3.92 (±0.03) ^a^	−3.21 (±0.28) ^b^	11.03
1.2 TFMF	64.22 (±0.05) ^a^	−3.94 (±0.05) ^a^	−0.28 (±0.17) ^a^	0.31
1.4 CFMF	64.71 (±0.08) ^a^	−3.89 (±0.03) ^a^	−0.33 (±0.04) ^a^	0.10
PEF-L	64.26 (±0.68) ^a^	−3.88 (±0.02) ^a^	−0.20 (±0.17) ^a^	0.25
PEF-M	64.58 (±0.51) ^a^	−3.88 (±0.01) ^a^	−0.16 (±0.26) ^a^	0.10
PEF-H	64.85 (±0.60) ^a^	−3.96 (±0.05) ^a^	−0.07 (±0.16) ^a^	0.37
PEF-L/0.65 TFMF	53.07 (±0.65) ^b^	−3.99 (±0.04) ^a^	−3.12 (±0.11) ^b^	11.81
PEF-M/0.65 TFMF	53.37 (±1.23) ^b^	−3.99 (±0.04) ^a^	−3.52 (±0.03) ^b^	11.63
PEF-H/0.65 TFMF	53.44 (±1.38) ^b^	−4.02 (±0.04) ^a^	−3.28 (±0.24) ^b^	11.49
PEF-L/1.2 TFMF	64.51 (±0.50) ^a^	−3.92 (±0.02) ^a^	−0.15 (±0.22) ^a^	0.02
PEF-M/1.2 TFMF	64.36 (±0.42) ^a^	−3.92 (±0.01) ^a^	−0.23 (±0.03) ^a^	0.15
PEF-H/1.2 TFMF	64.65 (±0.32) ^a^	−4.00 (±0.02) ^a^	−0.20 (±0.06) ^a^	0.18
PEF-M/1.4 CFMF	64.57 (±0.18) ^a^	−3.91 (±0.02) ^a^	−0.34 (±0.07) ^a^	0.20
*P* ^1^	*	NS	*	-
SEM ^2^	0.539	0.010	0.148	0.711

^a,^ ^b^ Different superscripted letters in the same column indicate statistical significance between the two means. Values in parentheses following the mean values of the color attributes indicate the standard deviations. ^1^ *P* stands for the statistical probability. * Refers to a statistical significance of *p* < 0.05. NS indicates no statistical significance. ^2^ SEM abbreviates the standard error of the mean.

Total color difference, Δ*E*, representing an overall color difference of treated samples from the untreated skim milk was calculated using equation 1 and presented in Table 1. Color difference classification was adopted from Cserhalmi and others [27]. Based on this classification system Δ*E* can be categorized as: 0 to 0.5 = “not noticeable”, 0.5 to 1.5 = “slightly noticeable” and >1.5 = “noticeable”. Slightly noticeable color differences of 0.51 and 0.79 were observed in skim milk treated with HTST treatment at 75 °C or 95 °C, respectively. However, a noticeable Δ*E* of 11.03 was obtained in skim milk processed with MF-0.65 (*p* < 0.05). Similar values (*p* ≥ 0.05) were obtained when combination treatments were applied consisting of PEF (PEF-L, PEF-M, and PEF-H) and subsequent MF-0.65 for hurdle treatment of skim milk, yielding Δ*E*s of 11.83, 11.63 and 11.49, respectively. Overall, noticeably better color retention was achieved with PEF-L, PEF-M and PEF-H treatments; 1.2 µm TFMF and 1.4 µm CFMF-based processing, achieving lower Δ*E* values (in the range between 0.10 and 0.37 (*p* < 0.05)) than by the HTST and 0.65 µm TFMF treatments.

**Figure 1:**
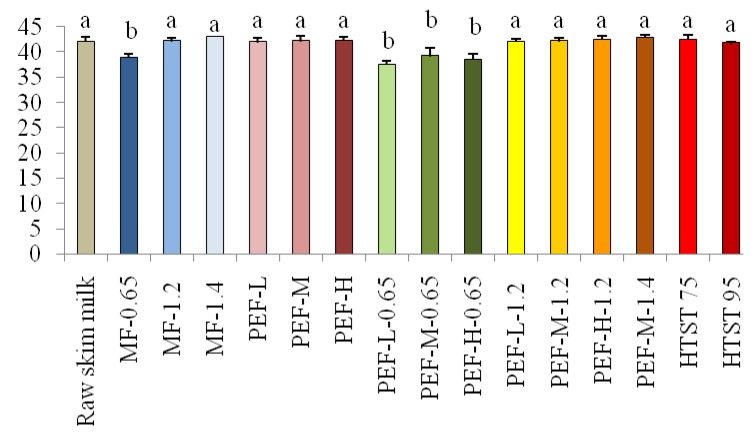
Whiteness obtained in untreated raw skim milk and by processing the latter with low-intensity (PEF-L), medium-intensity (PEF-M) and high-intensity (PEF-H) pulsed electric field, 0.65 (MF-0.65), 1.2 (MF-1.2), and 1.4 (MF-1.4) μm microfiltration, high-temperature short-time (HTST) pasteurization treatments and different pulsed electric field-based combinations with microfiltration treatments (after conversion from Hunter Lab color space data to CIE 1931 XYZ color space values). Different letters (*i.e.*, *a*, *b*) above bars indicate significant differences between treatment means (*p* < 0.05).

While skim milk pasteurized at 95 °C showed a positive yellowness value, no significant difference in any of the color attributes was observed (*p* ≥ 0.05) between the heat-treated skim milk compared to that processed by PEF, MF-1.2 and MF-1.4, and their combinations. Lightness was 16%–18% lower for all treatments that involved skim milk processing through 0.65 µm MF and the corresponding samples also exhibited a higher blueness as indicated by a lower *b* value (*p* < 0.05). As a result significantly lower whiteness index values were obtained for MF-0.65 and hurdle treatments combining PEF of different processing intensities with MF-0.65 as shown in Figure 1 (*p* < 0.05).

For the remaining non-thermal or thermal treatments no significant differences in whiteness were determined when compared among one another or with raw skim milk (*p* ≥ 0.05). Overall, non-thermal treatments using MF-1.2 MF-1.4 and PEF-L, PEF-M, PEF-H, and heat pasteurization did not have a significant impact on the single color properties of skim milk but, with the exception of MF-0.65-based treatments, thermally pasteurized skim milk was in a higher Δ*E* category than non-thermally treated skim milk.

### 3.2. Flavor Attributes of Non-Thermally and Thermally Treated Skim Milk

The effect of heat, pulsed electric field and microfiltration treatments on the volatile profile of skim milk was measured using SIFT-MS. Volatile compounds generated as a result of heat treatment and non-enzymatic browning reactions in skim milk were of main interest and, therefore, 21 compounds were selected for this study. Quantities of acetic acid and acetaldehyde were not detected by the mass spectrometer, so that data for 19 compounds (Table 2, Table 3 and Table 4) was obtained. Concentrations of ketones were found to be significantly higher in skim milk treated with HTST at 95 °C for a holding time of 45 s when compared to other treatments (*p* < 0.05). The major ketones contributing to this were 2-hexanone, 2-heptanone, 2-octanone, acetone, and butanone. However, there was no marked increase in concentration of these ketones in skim milk pasteurized at 75 °C for 20 s, nor for PEF-treated and MF-treated skim milk (*p* ≥ 0.05).

The aldehydes in skim milk were altered in a different way as compared to ketones in skim milk. Thermal treatments had a significant impact on 3-methylbutanal levels. The concentration of 3-methylbutanal was increased significantly upon heat treatment at 95 °C (*p* < 0.05). By contrast, microfiltration did not have an impact on aldehyde concentrations in skim milk. HTST treatments at 75 and 95 °C, PEF and microfiltration treatments did not have a significant impact on the concentration of heptanal or decanal (*p* ≥ 0.05).

Short chain fatty acids were the other group of volatiles analyzed in skim milk. Of these, hexanoic acid and butanoic acid were detected in raw skim milk, heat-treated, PEF-treated and microfiltered skim milk. Increased intensity of thermal treatments resulted in an increased concentration of hexanoic acid and the values were significantly higher in skim milk subjected to both HTST-75 and HTST-95 (*p* < 0.05) than in raw skim milk. Pasteurization at the higher temperature (HTST-95) led to a significant increase (*p* < 0.05) in the concentration of butanoic acid, whereas no impact (*p* ≥ 0.05) on its concentration was found for skim milk pasteurized at 75 °C. Moreover, no significant difference (*p* ≥ 0.05) in hexanoic and butanoic acid concentrations was observed between raw, PEF, MF, and hurdle treated skim milk.

The concentrations of both ethanol and propanol increased in skim milk following heat pasteurization at 95 °C (*p* < 0.05). Contrasting results (*p* ≥ 0.05) were obtained for PEF, MF, hurdle processing and HTST-75 treatments, indicating that their concentrations did not change in skim milk subjected to these processing conditions. Heat treatment at 95 °C resulted in increased levels of toluene (*p* < 0.05), while there was no significant impact by HTST-75, PEF, and MF treatments on this volatile compound (*p* ≥ 0.05).

The methyl acetate concentration was significantly higher (*p* < 0.05) in skim milk processed at 95 °C. None of the other processes affected the concentration of this volatile component (*p* ≥ 0.05). Furthermore, two sulfur compounds, hydrogen sulfide and dimethyl sulfide, were detected by the SIFT-MS equipment. Hydrogen sulfide concentrations were found to be at significantly higher levels in HTST-75 and HTST-95 treated skim milk, reaching the highest levels with the latter treatment (*p* < 0.05). The highest concentration of dimethyl sulfide occurred in skim milk that underwent the HTST-95 treatment. PEF processing and microfiltration did not increase the content of sulfur compounds.

**Table 2:** Retention of in 3-methyl butanal, heptanal, decanal, butanoic acid, hexanoic acid, dimethyl sulfide, and hydrogen sulfide obtained in skim milk after high-temperature short-time pasteurization (HTST_75_ = 75 °C for 20 s; HTST_95_ = 95 °C for 45 s) and lab-scale tangential-flow microfiltration (TFMF_0.65_ = 0.65 µm; TFMF_1.2_ = 1.2 µm), pilot scale cross-flow microfiltration (CFMF_1.4_ = 1.4 µm), pulsed electric field at low (PEF-L: 28 kV/cm; 2805 µs), moderate (PEF-M: 40 kV/cm, 1122 µs), and high (PEF-H: 40 kV/cm, 1571 µs) processing intensities and selected non-thermal combination treatments using PEF and subsequent microfiltration.

Treatments	3-Methyl butanal [ppb]	Heptanal [ppb]	Decanal [ppb]	Butanoic acid [ppb]	Hexanoic acid [ppb]	Dimethyl sulfide [ppb]	Hydrogen sulfide [ppb]
Raw Skim Milk	3.6 (±1.1) ^a^	15 (±6.2) ^a^	2.7 (±0.5) ^a^	14 (±6.0) ^a^	2.3 (±0.8) ^a^	12 (±5.9) ^a^	1.4 (±0.7) ^a,b^
HTST_75_	3.3 (±0.1) ^a^	15 (±1.9) ^a^	3.3 (±0.0) ^a^	13 (±1.4) ^a^	9.7 (±0.3) ^d^	17 (±2.2) ^a,b^	4.2 (±0.1) ^c^
HTST_95_	6.3 (±0.7) ^b^	24 (±5.2) ^a^	4.8 (±1.6) ^a^	44 (±6.0) ^b^	4.8 (±0.2) ^c^	25 (±6.1) ^b^	5.5 (±0.2) ^d^
TFMF_0.65_	3.9 (±0.0) ^a^	14 (±10.3) ^a^	2.2 (±0.3) ^a^	15 (±5.8) ^a,b^	1.1 (±0.3) ^a^	11 (±0.7) ^a,b^	n.d.
TFMF_1.2_	4.5 (±1.7) ^a^	17 (±7.3) ^a^	3.4 (±1.4) ^a^	13 (±1.8) ^a^	2.4 (±0.2) ^a,b^	12 (±0.9) ^a,b^	1.1 (±0.5) ^a,b^
CFMF_1.4_	3.5 (±1.5) ^a^	11 (±1.8) ^a^	2.3 (±0.8) ^a^	11 (±3.1) ^a^	2.5 (±0.3) ^a,b^	11 (±0.5) ^a,b^	1.1 (±0.4) ^a,b^
PEF-L	2.4 (±0.4) ^a^	10 (±0.7) ^a^	2.5 (±0.7) ^a^	12 (±3.3) ^a^	1.6 (±0.3) ^a,b^	9.1 (±3.4) ^a^	0.8 (±0.1) ^a^
PEF-M	3.0 (±1.7) ^a^	17 (±2.9) ^a^	3.4 (±0.8) ^a^	16 (±7.2) ^a,b^	1.8 (±0.5) ^a,b^	15 (±7.0) ^a,b^	1.3 (±0.2) ^a, b^
PEF-H	3.9 (±2.8) ^a^	14 (±2.2) ^a^	2.6 (±1.1) ^a^	15 (±5.1) ^a,b^	2.7 (±0.1) ^b^	18 (±2.1) ^a,b^	2.1 (±0.1) ^b^
PEF-L/TFMF_0.65_	2.5 (±0.4) ^a^	20 (±0.7) ^a^	3.9 (±2.3) ^a^	10 (±3.4) ^a^	1.7 (±0.2) ^a,b^	5.0 (±0.8) ^a^	1.1 (±0.3) ^a,b^
PEF-M/TFMF_0.65_	3.6 (±2.8) ^a^	21 (±1.0) ^a^	3.3 (±1.6) ^a^	13 (±5.2) ^a^	1.7 (±0.5) ^a,b^	7.5 (±1.3) ^a^	0.9 (±0.3) ^a,b^
PEF-H/TFMF_0.65_	4.8 (±2.5) ^a^	16 (±2.1) ^a^	3.2 (±0.5) ^a^	9.1 (±2.3) ^a^	1.5 (±0.5) ^a,b^	7.9 (±0.7) ^a^	1.1 (±0.2) ^a,b^
PEF-L/TFMF_1.2_	2.1 (±0.5) ^a^	14 (±2.7) ^a^	2.5 (±0.1) ^a^	8.6 (±1.9) ^a^	1.7 (±0.1) ^a,b^	9.2 (±1.9) ^a^	0.9 (±0.2) ^a,b^
PEF-M/TFMF_1.2_	5.6 (±0.2) ^a^	20 (±0.4) ^a^	1.5 (±1.2) ^a^	16 (±0.3) ^a,b^	2.5 (±0.1) ^a,b^	17 (±2.7) ^a,b^	1.6 (±0.2) ^a,b^
PEF-H/TFMF_1.2_	2.7 (±1.3) ^a^	13 (±3.0) ^a^	1.8 (±0.2) ^a^	8.9 (±3.2) ^a^	2.1 (±0.5) ^a,b^	12 (±2.4) ^a,b^	1.2 (±0.3) ^a,b^
PEF-M/CFMF_1.4_	2.0 (±0.2) ^a^	17 (±1.2) ^a^	3.5 (±0.1) ^a^	9.1 (±0.7) ^a^	1.4 (±0.4) ^a,b^	14 (±2.3) ^a,b^	1.0 (±0.0) ^a^^,b^
*P* ^1^	*	NS	NS	*	*	*	*
SEM ^2^	0.19	0.64	0.12	1.53	0.20	0.87	0.11

^a,^ ^b,^ ^c^ Different superscripted letters in the same column indicate statistical significance between the two means. Values in parentheses following the mean values of compound indicate the standard deviations. ^1^ *P* stands for the statistical probability. * Refers to a statistical significance of *p* < 0.05. NS indicates no statistical significance. ^2^ SEM abbreviates the standard error of the mean.

**Table 3:** Retention of in ethanol, 1-propanol, 1-octene, toluene, *p*-xylene, and methyl acetate obtained in skim milk after high-temperature short-time pasteurization (HTST_75_ = 75 °C for 20 s; HTST_95_ = 95 °C for 45 s) and lab-scale tangential-flow microfiltration (TFMF_0.65_ = 0.65 µm; TFMF_1.2_ = 1.2 µm), pilot scale cross-flow microfiltration (CFMF_1.4_ = 1.4 µm), pulsed electric field at low (PEF-L: 28 kV/cm; 2805 µs), moderate (PEF-M: 40 kV/cm, 1122 µs), and high (PEF-H: 40 kV/cm, 1571 µs) processing intensities and selected non-thermal combination treatments using PEF and subsequent microfiltration.

Treatments	Ethanol [ppb]	1-Propanol [ppb]	1-Octene [ppb]	Toluene [ppb]	*p*-Xylene [ppb]	Methyl acetate [ppb]
Raw Skim Milk	65 (±18) ^a^	10 (±2.0) ^a^	34 (±8.3) ^a^	4.2 (±0.8) ^a^	1.6 (±0.9) ^a^	6.7 (±1.3) ^a^
HTST_75_	57 (±9.9) ^a,b^	11 (±1.8) ^a,b^	31 (±2.1) ^a^	4.6 (±0.8) ^a^	1.5 (±0.3) ^a^	10 (±3.0) ^a,b^
HTST_95_	131 (±1.8) ^c^	16 (±2.1) ^b^	38 (±1.5) ^a^	6.9 (±1.6) ^b^	2.0 (±0.8) ^a^	19 (±4.1) ^b^
TFMF_0.65_	51 (±6.0) ^a,b^	11 (±0.8) ^a,b^	15 (±1.7) ^a^	4.0 (±0.7) ^a^	1.6 (±1.1) ^a^	6.7 (±0.8) ^a,b^
TFMF_1.2_	60 (±0.9) ^a,b^	10 (±1.3) ^a,b^	33 (±8.3) ^a^	4.6 (±0.6) ^a^	1.3 (±0.3) ^a^	6.3 (±0.5) ^a,b^
CFMF_1.4_	61 (±27) ^a,b^	9.2 (±3.3) ^a,b^	22 (±3.7) ^a^	4.5 (±1.8) ^a^	2.7 (±0.5) ^a^	5.6 (±2.3) ^a,b^
PEF-L	39 (±2.0) ^a^	7.1 (±1.8) ^a,b^	28 (±7.8) ^a^	2.8 (±0.2) ^a^	0.9 (±0.5) ^a^	3.5 (±0.1) ^a^
PEF-M	64 (±16) ^a,b^	9.3 (±2.8) ^a,b^	39 (±8.0) ^a^	5.5 (±0.2) ^a^	1.4 (±0.4) ^a^	7.8 (±3.1) ^a,b^
PEF-H	61 (±7.1) ^a,b^	9.6 (±4.3) ^a,b^	29 (±4.7) ^a^	2.4 (±0.1) ^a^	2.5 (±0.2) ^a^	7.5 (±0.7) ^a,b^
PEF-L/TFMF_0.65_	18 (±13) ^a^	4.8 (±0.2) ^a^	31 (±11) ^a^	2.6 (±0.0) ^a^	1.9 (±0.0) ^a^	5.4 (±0.4) ^a^
PEF-M/TFMF_0.65_	39 (±36) ^a^	6.1 (±1.7) ^a,b^	26 (±5.7) ^a^	3.9 (±0.3) ^a^	2.1 (±0.6) ^a^	3.8 (±0.4) ^a^
PEF-H/TFMF_0.65_	44 (±2.9) ^a^	5.2 (±2.2) ^a^	24 (±3.4) ^a^	2.2 (±0.2) ^a^	1.2 (±0.5) ^a^	5.2 (±0.5) ^a^
PEF-L/TFMF_1.2_	58 (±2.6) ^a,b^	5.2 (±0.1) ^a^	28 (±7.7) ^a^	2.0 (±0.4) ^a^	0.9 (±0.3) ^a^	4.1 (±0.6) ^a^
PEF-M/TFMF_1.2_	84 (±5.3) ^b,c^	12 (±0.8) ^a,b^	37 (±1.8) ^a^	5.6 (±0.7) ^a^	3.0 (±1.1) ^a^	6.4 (±0.5) ^a,b^
PEF-H/TFMF_1.2_	52 (±3.4) ^a,b^	7.4 (±3.2) ^a^	29 (±2.2) ^a^	3.0 (±0.9) ^a^	1.1 (±0.6) ^a^	3.8 (±2.2) ^a^
PEF-M/CFMF_1.4_	42 (±12) ^a,b^	6.6 (±1.5) ^a,b^	25 (±5.8) ^a^	2.2 (±0.4) ^a^	1.5 (±0.1) ^a^	4.1 (±0.1) ^a^
*P* ^1^	*	*	N.S.	*	N.S.	*
SEM ^2^	3.06	0.34	0.95	0.17	0.13	0.37

^a,^ ^b,^ ^c^ Different superscripted letters in the same column indicate statistical significance between the two means. Values in parentheses following the mean values of compound indicate the standard deviations. ^1^ *P* stands for the statistical probability. * Refers to a statistical significance of *p* < 0.05. NS indicates no statistical significance. ^2^ SEM abbreviates the standard error of the mean.

**Table 4:** Retention of in acetone, butanone, 2-hexanone, 2-heptanone, 2-octanone and 2-nonanone obtained in skim milk after high temperature short time pasteurization (HTST_75_ = 75 °C for 20 s; HTST_95_ = 95 °C for 45 s) and lab-scale tangential-flow microfiltration (TFMF_0.65_ = 0.65 µm; TFMF_1.2_ = 1.2 µm), pilot scale cross-flow microfiltration (CFMF_1.4_ = 1.4 µm), pulsed electric field at low (PEF-L: 28 kV/cm; 2805 µs), moderate (PEF-M: 40 kV/cm, 1122 µs), and high (PEF-H: 40 kV/cm, 1571 µs) processing intensities and selected non-thermal combination treatments using PEF and subsequent the microfiltration.

Treatments	Acetone [ppb]	Butanone [ppm]	2-Hexanone [ppb]	2-Heptanone [ppb]	2-Octanone [ppb]	2-Nonanone [ppb]
Raw Skim Milk	2.7 (± 0.8) ^a^	174 (±51) ^a^	6.8 (±3.1) ^a^	4.9 (±2.5) ^a^	9.7 (±6.5) ^a^	4.2 (±1.1) ^a^
HTST_75_	4.0 (±0.4) ^a, b^	225 (±23) ^a, b^	9.0 (±1.1) ^a, b^	4.8 (±1.7) ^a, b^	11 (±1.8) ^a, b^	5.5 (±0.8) ^a^
HTST_95_	6.2 (±1.0) ^b^	344 (±0.7) ^b^	9.3 (±0.3) ^b^	12 (±2.6) ^b^	16 (±3.6) ^b^	5.9 (±0.3) ^a^
TFMF_0.65_	2.4 (±0.4) ^a^	169 (±23) ^a^	2.8 (±1.6) ^a^	4.6 (±1.1) ^a, b^	8.9 (±1.4) ^a, b^	3.3 (±0.1) ^a^
TFMF_1.2_	2.6 (±0.2) ^a, b^	188 (±56) ^a^	3.4 (±1.8) ^a, b^	4.9 (±4.0) ^a, b^	9.5 (±3.5) ^a, b^	3.7 (±2.7) ^a^
CFMF_1.4_	2.3 (±0.9) ^a^	140 (±51) ^a^	7.0 (±1.6) ^a, b^	3.7 (±0.3) ^a, b^	6.5 (±0.6) ^a^	5.0 (±2.4) ^a^
PEF-L	1.8 (±1.0) ^a^	120 (±32) ^a^	5.7 (±1.5) ^a, b^	3.9 (±0.2) ^a, b^	7.2 (±1.4) ^a, b^	2.6 (±0.4) ^a^
PEF-M	2.6 (±1.9) ^a, b^	135 (±74) ^a^	7.0 (±0.7) ^a, b^	5.1 (±2.8) ^a, b^	8.8 (±3.5) ^a, b^	3.5 (±1.9) ^a^
PEF-H	3.1 (±2.2) ^a, b^	147 (±58) ^a^	7.8 (±0.3) ^a, b^	4.8 (±2.2) ^a, b^	7.9 (±4.2) ^a, b^	5.4 (±0.9) ^a^
PEF-L/TFMF_0.65_	1.5 (±0.9) ^a^	54 (±8.0) ^a^	5.0 (±0.1) ^a, b^	6.0 (±1.2) ^a, b^	8.7 (±1.8) ^a, b^	5.2 (±3.8) ^a^
PEF-M/TFMF_0.65_	1.9 (±1.1) ^a^	75 (±40) ^a^	5.7 (±0.2) ^a, b^	6.5 (±1.4) ^a, b^	8.8 (±1.8) ^a, b^	5.4 (±3.6) ^a^
PEF-H/TFMF_0.65_	1.9 (±1.1) ^a^	80 (±41) ^a^	6.9 (±0.6) ^a, b^	4.7 (±3.1) ^a, b^	7.4 (±2.8) ^a, b^	4.5 (±0.9) ^a^
PEF-L/TFMF_1.2_	1.5 (±0.4) ^a^	56 (±18) ^a^	5.0 (±0.1) ^a, b^	3.5 (±0.3) ^a^	7.5 (±0.3) ^a, b^	1.9 (±0.5) ^a^
PEF-M/TFMF_1.2_	3.0 (±0.2) ^a, b^	74 (±26) ^a^	7.6 (±0.4) ^a, b^	6.6 (±1.0) ^a, b^	12 (±0.0) ^a, b^	5.7 (±0.4) ^a^
PEF-H/TFMF_1.2_	2.2 (±0.6) ^a^	84 (±60) ^a^	5.4 (±1.3) ^a, b^	4.8 (±1.2) ^a, b^	8.3 (±0.9) ^a, b^	2.9 (±0.1) ^a^
PEF-M/CFMF_1.4_	2.1 (±0.4) ^a^	81 (±11) ^a^	8.9 (±0.2) ^b^	3.4 (±0.3) ^a^	8.8 (±2.4) ^a^	4.0 (±0.7) ^a^
*P* ^1^	*	*	*	*	*	NS
SEM ^2^	0.12	8.01	0.38	0.36	0.54	0.23

^a,^ ^b,^ ^c^ Different superscripted letters in the same column indicate statistical significance between the two means. Values in parentheses following the mean values of compound indicate the standard deviations. ^1^ *P* stands for the statistical probability. * Refers to a statistical significance of *p* < 0.05. NS indicates no statistical significance. ^2^ SEM abbreviates the standard error of the mean.

## 4. Discussion

For Hunter *L*, *a*, *b* color space values, no significant differences were observed in lightness, redness, and yellowness for skim milk treated with PEF, HTST and, MF-1.2 and MF-1.4 (*p* ≥ 0.05). A significant reduction in *L*, *b* and whiteness index values for skim milk that was microfiltered through a 0.65 µm pore size membrane indicates that higher cut-off membranes partially retain suspended milk components [28,29]. Pafylias *et al.* [30] suggested the use of larger pore size membranes of about 1.4 µm to maintain a balance between reduction of bacterial cells and retention of milk components. However, we did not see any significant differences in color attributes for skim milk processed through 1.2 and 1.4 µm pore size MF. Silva *et al.* [31] compared instrumental color differences between microfiltered and pasteurized skim milk and found smaller changes in color coordinates of microfiltered skim milk due to a lack of reactions caused by heating skim milk to pasteurization temperatures. HTST treatments caused a small increase in lightness values for all the samples, which may be due to an increase in number of dispersed components as a result of denaturation of milk proteins at high temperatures [20]. However, the increase in lightness was not significant (*p* ≥ 0.05). The Hunter-*b* value was positive for HTST-95 treatment indicating yellowness in skim milk. This might be attributed to heat induced browning reactions in milk between lactose and amino acids [32,33,34]. However, PEF and MF-treated skim milk retained milk color better than heat treatments as indicated by “not noticeable” total color difference values compared to raw skim milk. As indicated by ∆*E* values, there was a lower impact on color of skim milk produced with the non-thermal technologies, with the exception of the smallest pore size MF that produced “unacceptable” color values. However, our results are not in agreement with results of Bermúdez-Aguirre *et al.* [35] who observed significant changes in *L*, *a*, *b* values of PEF-treated skim milk. The authors, however, did not observe any trends in color change and attributed this change partly to the wearing down of the electrode due to arcing.

Among the 21 volatile compounds analyzed, ketones represented a major class based on their expected contribution to flavor. Ketones in milk can originate from different sources. Some of them may be present in raw milk as a result of feed, e.g., acetone and 2-butanone, while others may be generated or accumulated as a result of β-oxidation of saturated fatty acids followed by decarboxylation or by β-ketoacid decarboxylation [36,37]. A significant increase in concentration of all methyl ketones, except 2-nonanone, was observed for samples subjected to heat treatment (*p* < 0.05). Increased levels of methyl ketones in milk due to thermal treatments have also been reported by other researchers [23,38]. The methyl ketone content of skim milk treated with either PEF or MF alone was similar to that of raw skim milk (*p* ≥ 0.05), indicating that they have a minimum effect on the concentration of lipids in skim milk. Comparable results (*p* ≥ 0.05) were obtained by hurdle processing of skim milk using both PEF and MF, suggesting a non-significant impact of combined processes on ketones in skim milk.

Another important class of compounds identified and relevant to this study were the sulfur compounds: dimethyl and hydrogen sulfide. These compounds are formed in milk as a result of thermal denaturation of milk whey proteins during processing and are responsible for cooked flavors in milk. Hydrogen sulfide is produced mainly from Strecker degradation of sulfhydryl groups of sulfur-containing amino acids (*i.e.*, cysteine and methionine) in whey proteins. Its production is also attributed to denatured proteins associated with the milk fat globule membrane and the rearrangement of a thiazole group during thermal degradation of thiamine [36,39,40]. In our research, the concentration of hydrogen sulfide in raw skim milk was found to be 1.39 ± 0.68 µg/L. Its concentration was higher when skim milk was pasteurized at 95 °C for 45 s (5.53 µg/L) as compared to skim milk subjected to 75 °C for 20 s (4.21 µg/L). Thus, the hydrogen sulfide concentration was found to increase with the severity of heat treatment. In contrast, PEF treatment applied at the highest intensity, which is known to generate increased temperature in the treatment chamber to about 65 °C by means of Joule heating, however, did not cause any significant increase in hydrogen sulfide concentration (2.05 µg/L) (*p* ≥ 0.05). The PEF-L treatment that resulted in the longest exposure (2805 µs) of skim milk to electric pulses also did not cause any significant changes in hydrogen sulfide concentration (0.82 µg/L) when compared to raw skim milk (*p* ≥ 0.05). The increase in concentration of sulfur compounds has been associated with growing denaturation of sulfur containing polypeptides affected to a large degree by increasing exposure to heat [39,41]. Dimethyl sulfide has been reported to occur naturally in cow’s milk as a result of diet and at low levels it contributes to milk flavor but at high concentrations it may impart an off-flavor to the milk [42,43]. It can also be formed as a result of thermal denaturation of sulfur containing amino acids in milk. Increased dimethyl sulfide content in milk subjected to heat treatment at pasteurization temperatures and ultra-high-temperatures has been reported by different research groups [22,44,45]. In this study, dimethyl sulfide concentration was observed to be significantly higher in skim milk processed at 95 °C for 45 s (23.6 µg/L) as compared to raw skim milk (6.17 to 17.95 µg/L). However, no significant differences in concentrations of dimethyl sulfide from those in raw skim milk were observed for milk that underwent HTST-75, PEF, and MF treatments (*p* ≥ 0.05). Hurdle processing of PEF with MF also did not cause any significant changes in concentrations of sulfur compounds in comparison to raw skim milk (*p* ≥ 0.05).

Among aldehydes, acetaldehyde concentrations were below the detection limit of SIFT-MS for raw as well as treated skim milk. Aliphatic aldehydes are produced as a result of auto-oxidation of unsaturated fatty acids as well as by breakdown of amino acids in milk [37,40]. No significant differences were found in heptanal and decanal concentrations among raw skim milk, HTST-treated, PEF-treated, and MF-treated skim milk (*p* ≥ 0.05). In contrast, Zhang and others [5] observed significantly increased heptanal and decanal levels in pasteurized milk in comparison to raw milk while no significant differences were found in PEF-treated skim milk. However, the increase in aldehyde concentrations in skim milk upon exposure to heat treatment at 95 °C, though not significant (*p* ≥ 0.05) could contribute to significant changes in aroma of skim milk due to lower threshold values for some aldehydes [44]. 3-Methyl butanal is a product of Strecker degradation of leucine during the non-enzymatic browning reaction, which occurs during heat treatment of milk [36,37]. No significant increase in its concentration was found after non-thermal PEF and MF treatments (*p* ≥ 0.05). Its presence in skim milk treated at 95 °C was found to be significantly higher (*p* < 0.05) than in raw skim milk and in skim milk processed by all other treatments tested. Contarini and Povolo [23] also observed a positive correlation between increased levels of 3-methyl butanal and severity of heat treatment, while no significant differences were found in heptanal concentrations.

Short-chain and medium-chain fatty acids (C-4 to C-14 and some C-16) comprise 45% of total fatty acids in milk fat and are produced in the mammary gland from acetate and β-hydroxybutyrate, the products of bacterial fermentation in the rumen. Activation of acetyl to acetyl CoA and its carboxylation to malonyl CoA results in a stepwise addition of two CH_2_ groups and hence, increase in fatty acid chain length from short- to medium-chain fatty acids [46]. Out of these straight-chained, even numbered fatty acids, butanoic and hexanoic acids were quantified in this study. An increase in concentration of these two short-chain fatty acids as a result of heat exposure has been reported by Gandy and others [24]. Significantly higher amounts of both butanoic acid and hexanoic acid were observed in skim milk treated at 95 °C. However, HTST pasteurization at 75 °C showed a greater increase in hexanoic acid and, interestingly, the increase was twice the increase observed for the 95 °C treatment. Increased levels of butanoic and hexanoic acid in heat treated milk may impart cheesy, sour, and cream notes to milk as classified by Zhang and others [5] under the category of high olfactometric intensity odorants.

Formation of alcohols by reduction of corresponding carbonyl compounds [29] was observed for milk stored at refrigeration temperatures, where alcohol concentration increased as result of reduction of carbonyl compounds [30,47]. HTST-75, PEF, and MF treatments did not have any significant impact on the concentration of ethanol and 2-propanol in skim milk. Similarly, Zhang *et al.* [5] reported no marked change in concentrations of alcohols subsequent to PEF and HTST processing. However, at a processing temperature of 95 °C, a marked increase in concentration of both these alcohols was observed, which indicates some reducing reactions take place at this temperature. Toluene and *p*-xylene are considered as products of thermal degradation of β-carotene [48] and these compounds have been associated with heat treated milk [24,38,49]. Esters are formed by reaction of carboxylic acids with alcohols. Methyl acetate was the only ester quantified in this study and its concentration increased three-fold in HTST-95 treated skim milk. As observed in similar studies [3,50,51] non-thermal processing had a lesser impact on the concentration of volatile compounds in skim milk than thermal processing and indicates that skim milk of better quality results from non-thermal processing. Last but not least, it is worthy of mention that different scales (lab, pilot plant) of MF processing equipments (1.2 µm TFMF and 1.4 µm CFMF, respectively) used in this study indicated comparable effects on color and composition of volatiles in skim milk, thereby, also substantiating that different designs and materials of the MF membrane modules had no significant effect on these organoleptical quality parameters.

## 5. Conclusions

MF (1.2 and 1.4 µm) and PEF alone and in combination (hurdle technology) caused no significant changes to skim milk color and its volatile compound composition, thus, the hurdle approach can be regarded as promising for preservation of skim milk of very good sensory quality. No noticeable color differences from raw skim milk were observed by application of PEF, and MF with a pore size of 1.2 µm or above. Smaller pore size microfiltration (0.65 µm) was found to be unsatisfactory when applied alone or in combination with PEF, resulting in significant changes to skim milk color attributes. Different scale, membrane materials, and membrane designs of the MF systems showed no effect on color and volatile compounds. A significant increase in ketones, fatty acids, hydrocarbons and sulfur compounds was obtained following heat treatment of skim milk at the higher intensity treatment (95 °C, 45 s) applied in this study, while lower intensity heat treatment (75 °C, 20 s) also resulted in increased concentration of selected volatile compounds. However, increased PEF intensity did not significantly alter concentrations of volatile compounds. These findings indicate PEF/MF is an emerging technology with potential to produce skim milk of high sensory quality. Further research on this promising hurdle technology is envisaged such as the study of enzyme activity and vitamin retention in PEF/MF-treated skim milk as well as its application for processing other liquid dairy products.
